# FKBP5 expression in human adipose tissue: potential role in glucose and lipid metabolism, adipogenesis and type 2 diabetes

**DOI:** 10.1007/s12020-018-1674-5

**Published:** 2018-07-21

**Authors:** Cherno O. Sidibeh, Maria J. Pereira, Xesus M. Abalo, Gretha J. Boersma, Stanko Skrtic, Per Lundkvist, Petros Katsogiannos, Felix Hausch, Casimiro Castillejo-López, Jan W. Eriksson

**Affiliations:** 1 Department of Medical Sciences, Uppsala University, Uppsala, Sweden; 2 AstraZeneca R&D, Mölndal, Sweden; 3 Institute of Medicine, Sahlgrenska Academy, University of Gothenburg, Gothenburg, Sweden; 4 Institute of Organic Chemistry and Biochemistry, Technical University Darmstadt, Darmstadt, Germany

**Keywords:** Type 2 diabetes, Glucocorticoids, Insulin resistance, Adipose tissue, FKBP51, SAFit1

## Abstract

**Purpose:**

Here, we explore the involvement of FKBP51 in glucocorticoid-induced insulin resistance (IR) in human subcutaneous adipose tissue (SAT), including its potential role in type 2 diabetes (T2D). Moreover, we assess the metabolic effects of reducing the activity of FKBP51 using the specific inhibitor SAFit1.

**Methods:**

Human SAT was obtained by needle biopsies of the lower abdominal region. *FKBP5* gene expression was assessed in fresh SAT explants from a cohort of 20 T2D subjects group-wise matched by gender, age and BMI to 20 non-diabetic subjects. In addition, human SAT was obtained from non-diabetic volunteers (20F/9M). SAT was incubated for 24 h with or without the synthetic glucocorticoid dexamethasone and SAFit1. Incubated SAT was used to measure the glucose uptake rate in isolated adipocytes.

**Results:**

*FKBP5* gene expression levels in SAT positively correlated with several indices of IR as well as glucose area under the curve during oral glucose tolerance test (*r* = 0.33, *p* < 0.05). *FKBP5* gene expression levels tended to be higher in T2D subjects compared to non-diabetic subjects (*p* = 0.088). Moreover, *FKBP5* gene expression levels were found to inversely correlate with lipolytic, lipogenic and adipogenic genes. SAFit1 partly prevented the inhibitory effects of dexamethasone on glucose uptake.

**Conclusions:**

*FKBP5* gene expression in human SAT tends to be increased in T2D subjects and is related to elevated glucose levels. Moreover, *FKBP5* gene expression is inversely associated with the expression of lipolytic, lipogenic and adipogenic genes. SAFit1 can partly prevent glucose uptake impairment by glucocorticoids, suggesting that FKBP51 might be a key factor in glucocorticoid-induced IR.

## Introduction

Type 2 diabetes (T2D) is a metabolic condition with ever increasing global prevalence. The World Health Organization reported that in 2014, the overall prevalence of diabetes was 8.5% among adults [1], increasing from 4.7% since 1980. Obesity is strongly associated with a state of insulin resistance [2–4] where tissues and organs such as adipose tissue, skeletal muscle and liver inadequately respond to insulin [5]. Insulin resistance, in combination with beta cell dysfunction, is usually critical for the development and progression of T2D [6, 7]. It is therefore of strong clinical relevance to elucidate the underlying causes and mechanisms involved in obesity and insulin resistance.

Steroid hormones, such as glucocorticoids, can be used as tools to achieve a better understanding of the development of obesity and insulin resistance. Glucocorticoids have found their way to frequent clinical use because of their anti-inflammatory and immunosuppressive properties [8, 9]. However, high exogenous or endogenous levels are linked to several adverse metabolic effects including increased obesity, insulin resistance and onset of diabetes similar to T2D [10, 11]. We recently assessed glucocorticoid effects on gene expression in human adipose tissue in a microarray-based approach [12]. We found that *FKBP5*, the gene that codes for FK506 binding protein 51 (FKBP51), was highly upregulated by the synthetic glucocorticoid dexamethasone in human subcutaneous and omental adipose tissue [12]. *FKBP5* gene expression levels were also found to be associated with markers of insulin resistance [12].

FKBP51 is a protein that has mostly been studied in the field of psychiatric disorders including anxiety and depression [13, 14]. This is likely due to the key role of FKBP51 in the hypothalamic–pituitary–adrenal axis (HPA-axis) [15]. The HPA-axis has a feedback control system that regulates the release of glucocorticoids, e.g. in response to stress [15, 16]. However, glucocorticoids have systemic metabolic effects beyond the central nervous system, on organs such as the pancreas, skeletal muscle and adipose tissue [17]. In addition, FKBP51 has increasingly emerged as a player in metabolic regulation [18]. This includes a recent report where the rs1360780 polymorphism of the *FKBP5* gene has been associated with reduced weight loss following bariatric surgery [19]. Furthermore, FKBP51 has been shown to regulate Akt/protein kinase B (PKB) activity [20], thus indicating an association between FKBP51 and insulin resistance. Studies in *FKBP5* knockout mice have also proposed FKBP51 as a link between chronic stress and obesity. Mice lacking the *FKBP5* gene have reduced bodyweight compared to wildtype mice and are resistant to diet-induced obesity [21, 22]. Furthermore, FKBP51 has been reported to play a role in adipogenesis in mouse-derived cells [23, 24]. The role of FKBP51 in systemic metabolism is further supported by its higher expression levels in metabolically active tissues such as skeletal muscle and adipose tissue in comparison to other tissues [18, 25].

FKBP51 resides in the cellular cytosol where its principal function is to regulate the glucocorticoid response through interaction with the glucocorticoid receptor (GR) as part of its downstream signalling. The GR, also located in the cytosol, forms a complex made up of multiple co-factors and chaperones. These include heat shock protein 90 (Hsp90), p23, FKBP51 and FK506 binding protein 52 (FKBP52) [26]. FKBP51 and FKPB52 compete for the binding to the GR-associated HSP90. An FKBP51-associated GR-complex has a low affinity for glucocorticoids, whereas an FKBP52-associated GR-complex has a high affinity for glucocorticoids [27]. When a glucocorticoid diffuses through the cell membrane it binds to the FKBP52-associated GR-complex. This facilitates the translocation of the entire complex into the nucleus where it binds to specific DNA response elements triggering the activation or suppression of gene transcription. One of the principal genes that is upregulated in this chain of events is the FKBP51-coding gene. FKBP51 thereby regulates its own expression in what is referred to as an ultra-short negative feedback loop [26–29].

To date, there are very few studies of FKBP51 in metabolically active tissues. Here, we want to further expand upon our previous findings where we established a relationship between FKBP51, glucocorticoids, insulin resistance and the impairment of insulin-stimulated glucose uptake in human adipose tissue [12]. Moreover, we want to explore the role of FKBP51 in T2D with respect to adipose metabolism and function. This includes elucidating its potential role in adipogenesis [23] and providing insight into what role, if any, FKBP51 may have in lipid metabolism.

## Materials and methods

### Adipose tissue donors

#### Cohort 1

Human subcutaneous adipose tissue (SAT) was obtained from a cohort of 20 T2D subjects and 20 non-diabetic subjects, matched by gender (10F/10M), age (58 ± 9 vs 58 ± 11 years, respectively) and BMI (30.7 ± 4.9 vs 30.8 ± 4.6 kg/m^2^, respectively). Fasting blood samples, oral glucose tolerance test (OGTT) and SAT needle biopsies were performed as previously described [12, 30]. SAT was acquired by needle aspiration of the lower abdominal region after local dermal anaesthesia with lidocaine (Xylocain; AstraZeneca, Sweden). Part of the adipose tissue was snap frozen in liquid nitrogen, stored at −80 °C and used to assess gene expression levels of *FKBP5*, other glucocorticoid-regulated genes and genes involved in adipogenesis and glucose, lipid and energy metabolism. SAT and plasma were also used to measure fasting cortisone and cortisol levels. In addition, SAT was used to measure ex vivo glucose uptake and lipolysis in isolated adipocytes. All measurements were performed as previously described [30]. Clinical and biochemical characteristics of the subjects are shown in Table 1.

**Table 1:** Clinical characteristics of study participants

	Cohort 1				Cohort 2	
	ND		T2D			
	Average	Range	Average	Range	Average	Range
*N*	20 (10M, 10F)		20 (10M, 10F)		29 (20F, 9M)	
Age, years	58	34–72	58	41–71	46	18–72
BMI (kg/m^2^)	30.8	22.7–38.4	30.7	22.5–39.9	27.4	22.7–47.2
WHR	0.96	0.84–1.09	0.99	0.90–1.08	0.87	0.74–1.00
Sc adipocyte diameter (µm)	109.6	91.4–124.7	106.4	82.6–124.6	102.1	73.2–126.7
P-glucose (mmol/L)	6.0	4.9–7.3	8.2	6.1–11.5	5.7	4.6–6.7
Serum insulin (mU/L)	11.5	4.1–26.0	15.5	4.1–31.0	8.3	2.4–26.0
HOMA-IR	3.08	1.17–7.33	5.27	1.25–10.83	2.15	0.57–7.40
HbA_1c_, IFCC (mmol/mol)	37.3	31.0–46.0	48.8	37.0–73.0	32.8	22.0–40.0
HbA_1c_ (%)	5.6	5.0–6.4	6.6	5.5–8.8	5.2	4.2–6.6
P-total cholesterol (mmol/L)	5.7	4.4–8.4	4.9	3.1–6.8	5.0	3.0–7.2
P-HDL-cholesterol (mmol/L)	1.3	0.9–1.9	1.2	0.8–1.8	1.5	0.8–2.3
P-LDL-cholesterol (mmol/L)	3.6	1.9–6.0	3.1	1.8–4.9	3.0	1.0–5.2
P-triglycerides (mmol/L)	1.6	0.7–3.5	1.6	0.6–2.6	1.0	0.6–2.2

*ND* non-diabetic, *BMI* body mass index, *WHR* waist-hip ratio, *Sc* subcutaneous, *HOMA-IR* homoeostatic model assessment of insulin resistance, *HbA*_*1c*_ glycated haemoglobin, *P* plasma, *HDL* high-density lipoprotein, *LDL* low-density lipoprotein

#### Cohort 2

For the second cohort, the same procedure was used to extract SAT, in this case from non-diabetic volunteers (20F/9M, 18–72 years, BMI 22.7–47.2 kg/m^2^), and used to assess gene expression levels for *FKBP5* and other genes known to be regulated by the GR-complex (*n* = 9). In addition, SAT was used to isolate preadipocytes to investigate the role of FKBP51 in adipogenesis (*n* = 3). SAT samples were also used to study the effects of dexamethasone treatment and SAFit1, an FKBP51-specific inhibitor [31], on glucose uptake in isolated primary adipocytes (*n* = 19). Due to limited amounts of SAT obtained from biopsies, not all experiments were performed on samples from every subject.

Fasting blood samples were collected for analysis of plasma glucose, insulin and lipids at the Department of Clinical Chemistry at Uppsala University Hospital. Subjects with type 1 diabetes and/or T2D, other endocrine disorders, cancer or other major illnesses, as well as ongoing medication with beta-adrenergic blockers, systemic glucocorticoids or immune-modulating therapies were excluded from the study. Clinical and biochemical characteristics of the subjects are shown in Table 1.

The study protocols were approved by the Regional Ethics Review in Uppsala (Dnr 2013/330 and Dnr 2013-183/494). Written informed consent was obtained from all study participants.

### mRNA levels assessment

mRNA expression levels of *FKBP5*, other glucocorticoid-regulated genes and genes involved in adipogenesis and glucose, lipid and energy metabolism, in SAT from Cohort 1, were measured by RNAseq at Exiqon A/S, Vedbaek, Denmark, as previously described [30].

For real-time PCR assays, RNA was isolated from incubated SAT from Cohort 2 subjects or preadipocytes in culture using the RNeasy Lipid Tissue Mini Kit (Qiagen, Hilden, Germany) according to manufacturer’s protocol. Purified RNA was quantified using a NanoDrop ND-1000 spectrophotometer (NanoDrop Technologies, Wilmington, DE, USA). This was followed by conversion of RNA to cDNA using a high-capacity cDNA reverse transcription kit (Applied Biosystems, Foster City, CA, USA) and relative quantification using TaqMan probes towards target genes. See Supplementary Materials and Methods for details on real-time PCR assay.

### Adipose tissue incubation and assessments

The potency of glucocorticoids was assessed as their effects on β-adrenergic receptor expression (EC50 4.8 nmol/L for dexamethasone 24 nmol/L for cortisol). In this context, dexamethasone has been shown to elicit ~5 times the potency of cortisol (EC50 4.8 nmol/L for dexamethasone and 24 nmol/L for cortisol) [32]. Therefore a 0.3 µM concentration of dexamethasone would correspond to a maximum physiological plasma level of cortisol under stress conditions of about 1–2 µM [33].

SAT obtained from Cohort 1 subjects was instantaneously used to isolate primary adipocytes by collagenase (Sigma-Aldrich, St. Louis, MO, USA) treatment. Following isolation, primary adipocytes were washed in glucose-free Krebs-Ringer-Hepes medium or Hank's medium 199 for glucose uptake or lipolysis assays, respectively. Medium was supplemented with 4% bovine serum albumin and 150 nM adenosine. Following washing, glucose uptake and lipolysis assays were performed as previously described [12, 30]. See Supplementary Materials and Methods for additional details on the glucose uptake and lipolysis assays.

SAT from Cohort 2 was incubated in Dulbecco’s modified Eagle medium (DMEM, Invitrogen; 6 mM glucose, 10% fetal bovine serum (FBS), 1% penicillin–streptomycin (PEST)) with or without the glucocorticoid dexamethasone (0.3 µM) and with or without an FKBP5-selective small molecule inhibitor SAFit1 [33] (100, 500, 2000 or 10,000 nM concentrations) for 24 h (*n* = 7–19). Furthermore, SAT was incubated in DMEM (6 mM glucose, with 10% FBS, 1% PEST) for 24 h in 37 °C, 5% CO_2_ with or without SAFit1 alone (500 nM, *n* = 4). This was to assess the dependency of the effects of SAFit1 on the presence of glucocorticoids. Incubated SAT was used to test the effects of dexamethasone and the FKBP51 inhibitor on subsequent adipocyte glucose uptake as previously described [12, 30]. See Supplementary Materials and Methods for additional details on the glucose uptake assays.

Furthermore, part of the SAT was incubated with or without dexamethasone (0.3 µM) or dexamethasone together with SAFit1 for gene expression analyses (500 nM, *n* = 9).

### Stromal vascular fraction assessment

To assess the involvement of FKBP51 in human adipogenesis, the stromal vascular fraction (SVF), that contains preadipocytes, was isolated from human SAT. Fresh SAT from Cohort 2 was treated with collagenase (Sigma-Aldrich), to separate the SVF cells from the mature adipocytes, as previously described [12, 30]. The SVF was then cultured in preadipocyte medium until reaching a confluency of 70–80%. Cells were expanded until ~1,000,000 cells and transferred to 24-well plates at a density of 15,000 cells/cm^2^. Upon reaching 100% confluence, cells were differentiated as previously described [34, 35]. Preadipocytes were maintained in differentiation medium for a total of 14 days. Samples for gene and protein expression were collected on days 0, 7 and 14 (*n* = 5). In addition, Oil Red O staining and subsequent imaging of differentiating cells were performed on days 7 and 14 of differentiation (*n* = 3). See Supplementary Materials and Methods for more details regarding preadipocyte differentiation.

### Imaging and quantification of adipocyte differentiation

The lipids of the adipocytes undergoing ex-vivo differentiation were stained with Oil Red O solution on days 7 and 14 of differentiation. This was performed to quantify lipid accumulation and the rate of differentiation. See Supplementary Materials and Methods for details.

### Glucose uptake in adipocytes differentiated ex-vivo

Glucose uptake was assessed in ex-vivo differentiated human adipocytes (*n* = 4). The effects of dexamethasone on the glucose uptake rates of differentiated adipocytes were also assessed. On day 14 of differentiation, adipocytes were deprived of glucocorticoids for 48 h. On day 16, media was supplemented with 0.3 µM of dexamethasone for 24 h and on day 17, glucose uptake was performed as previously described [34, 36, 37]. In addition, samples for gene (*n* = 2) and protein (*n* = 4) expression were collected on day 17.

### Western blot

Protein lysates of cells in culture or homogenised adipose tissue were assessed using western blotting. In brief, blotting was performed by using equal amounts of protein for all samples (10 µg) and with the use of primary antibody to FKBP51 (ab126715, Abcam, Cambridge, UK; diluted 1:1000). Horseradish peroxidase-linked anti-rabbit IgG antibody (7074S, CST, diluted 1:2000) was used as a secondary antibody.

### Statistical analysis

All data are presented as mean ± SEM, unless stated otherwise. All statistical analyses were performed using IBM SPSS Statistics software. The Shapiro–Wilk test of normality was used to determine the normal distribution of data sets.

SAT gene expression data from Cohort 1 was log-transformed. Pearson’s bivariate correlation test was used to explore the associations between gene transcripts with respect to *FKBP5*. Spearman’s bivariate correlation test was used to assess correlations between *FKBP5* gene expression and metabolic parameters in Cohort 1. Significant variables were subsequently included in multivariate stepwise regression analyses.

Analyses of differences in gene and protein expression, differentiation rate and glucose uptake in Cohort 2 SAT and adipocytes differentiated ex vivo, were performed pairwise using the paired-samples *t* test.

Spearman’s bivariate correlation test was used to assess correlations of the percentage effects on glucose uptake by dexamethasone treatment vs dexamethasone and SAFit1 co-treatment in Cohort 2. A *p*-value < 0.05 was considered statistically significant.

## Results

### *FKBP5* expression levels in SAT

**Fig. 1:**
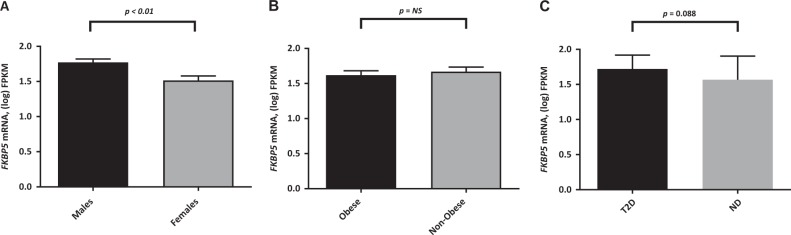
*FKBP5* gene expression is higher in SAT from male subjects compared to SAT from female subjects **a**. *FKBP5* gene expression levels in SAT do not differ between non-obese and obese subjects **b**. There was a tendency of higher *FKBP5* gene expression in SAT from T2D subjects compared to non-diabetic subjects **c**. *n* = 20, each group. ND non-diabetic, NS not significant

SAT from males in Cohort 1 had ~16% higher gene expression levels of *FKBP5* (*p* < 0.01) than females (Fig. 1a). There was also a tendency of higher *FKBP5* gene expression in SAT from T2D subjects (by 10%, *p* = 0.088) compared to healthy subjects (Fig. 1c). No differences were found between non-obese and obese subjects (Fig. 1b).

### Association between *FKBP5* gene expression levels in SAT and metabolic parameters

*FKBP5* gene expression levels positively correlated with markers of insulin resistance, including the glucose area under the curve (AUC) during OGTT (*r* = 0.33, *p* < 0.05), fasting glucose (*r* = 0.47, *p* < 0.01), Quantitative Insulin Sensitivity Check Index (QUICKI; r = -0.33, p<0.05) and HOMA-IR (*r* = 0.34, *p* < 0.05). *FKBP5* gene expression also correlated negatively with OGTT-derived insulin sensitivity indices, Matsuda (*r* = −0.34, *p* < 0.05) and Gutt (*r* = −0.32, *p* < 0.05). An association was also found between *FKBP5* gene expression and systolic blood pressure (SBP) (*r* = 0.32, *p* < 0.05). After inclusion of glucose AUC during OGTT, HOMA-IR, Matsuda index and SBP in a multivariate regression analysis; glucose AUC during OGTT (standard *β* coefficient = 0.32, *p* < 0.05; model: *r*^2^ = 0.27) remained the only significant predictor of *FKBP5* gene expression in SAT (Table 2). See Supplementary Results for additional details on associations between *FKBP5* gene expression and metabolic parameters.

**Table 2:** Association between *FKBP5* gene expression in SAT and metabolic parameters in Cohort 1 subjects (*n* = 40)

Metabolic parameters (Cohort 1)	*FKBP5* gene expression			
	Bivariate correlation^a^		Multivariate stepwise regression^b^	
	*r*	*p*	Std *β*	*p*
**AUC glucose**	0.33	0.039	**0.32**	**0.043**
**HOMA-IR**	0.34	0.033	–	NS
**Matsuda**	-0.34	0.034	–	NS
**SBP**	0.32	0.044	–	NS

*AUC* area under the curve, *HOMA-IR* homoeostatic model assessment of insulin resistance, *Matsuda* Matsuda insulin sensitivity index, *SBP* systolic blood pressure, *NS* not significant

^a^ *r*-Values are Spearman correlation coefficients. Variables with *p* value < 0.05 were considered to multivariate stepwise regression analysis

^b^ Only the variables that had a *p* value < 0.05 were considered in the final fitted model. Std *β* is the standard beta coefficient

### Expression of the *FKBP5* gene is associated with genes regulating lipolysis, glucose uptake and adipogenesis in SAT

SAT gene expression data from the Cohort 1 subjects revealed that *FKBP5* gene expression levels were associated with genes regulated by the GR. There were positive associations with *GR* (*r* = 0.45, *p* < 0.01) and *CNR1* (*r* = 0.50, *p* < 0.01) and inverse associations with *HSP90B1* (*r* = −0.39, *p* < 0.05) and *TIMP4* (*r* = −0.43, *p* < 0.01) (Table 3).

**Table 3:** Associations between *FKBP5* gene expression and various metabolic genes in subcutaneous adipose tissue from Cohort 1

	All subjects (*n* = 40)			ND (*n* = 20)		T2D (*n* = 20)		Male (*n* = 20)		Female (*n* = 20)	
	*r*		*p*	*r*	*p*	*r*	*p*	*r*	*p*	*r*	*p*
**GR-regulated genes**											
*HSD11B1*	0.07		0.686	0.19	0.420	0.06	0.803	−0.11	0.642	0.27	0.247
*GR*	**0.45**		**0.004**	**0.66**	**0.002**	0.12	0.620	**0.62**	**0.003**	0.25	0.290
*HSP90AA1*	0.20		0.206	0.34	0.144	0.15	0.537	0.18	0.452	0.31	0.177
*HSP90AB1*	0.12		0.481	0.19	0.431	0.06	0.818	0.08	0.726	0.12	0.617
*HSP90B1*	**−0.39**		**0.013**	−0.41	0.076	−0.21	0.368	−0.36	0.118	−0.44	0.053
*CNR1*	**0.50**		**0.001**	**0.54**	**0.015**	0.43	0.059	0.40	0.078	**0.64**	**0.002**
*GILZ*	0.27		0.089	0.26	0.268	0.35	0.129	0.03	0.892	0.16	0.515
*TIMP4*	**−0.43**		**0.006**	**−0.60**	**0.005**	−0.35	0.126	−0.32	0.165	−0.56	0.010
**Genes of inflammatory cytokines**											
*TNF*	−0.02		0.895	0.01	0.964	−0.15	0.526	−0.07	0.781	0.11	0.636
*IFNG*	0.05		0.758	−0.08	0.746	0.29	0.231	0.31	0.198	−0.33	0.161
**Genes of adipokines**											
*ADIPOQ*	0.06		0.730	0.36	0.117	−0.12	0.602	0.15	0.525	0.39	0.091
*LEP*	−0.28		0.084	−0.25	0.286	−0.30	0.206	−0.20	0.399	0.01	0.983
**Lipid metabolism genes**											
*PDE3B*	**0.60**	**0.000**		**0.62**	**0.004**	**0.49**	**0.030**	**0.64**	**0.002**	**0.65**	**0.002**
*ATGL*	**−0.50**	**0.001**		**−0.53**	**0.016**	**−0.47**	**0.035**	−0.40	0.083	**−0.56**	**0.011**
*MGLL*	**−0.51**	**0.001**		**−0.56**	**0.011**	**−0.50**	**0.025**	−0.31	0.177	−0.40	0.077
*LIPE*	−0.28	0.082		−0.09	0.696	−0.42	0.064	−0.23	0.321	−0.22	0.351
*PLIN1*	−0.26	0.107		−0.41	0.072	−0.27	0.338	−0.13	0.586	−0.48	0.031
*PLIN2*	0.05	0.772		0.23	0.325	0.33	0.154	−0.28	0.240	0.37	0.108
*PLIN3*	**−0.47**	**0.002**		0.39	0.087	**−0.49**	**0.029**	**−0.58**	**0.008**	−0.32	0.175
*PLIN4*	**−0.43**	**0.006**		**−0.50**	**0.026**	**−0.45**	**0.046**	−0.35	0.136	**−0.49**	**0.028**
*LPL*	−0.05	0.746		0.16	0.506	−0.15	0.543	0.10	0.674	0.18	0.462
*DGAT1*	**−0.62**	**0.000**		**−0.58**	**0.007**	**−0.60**	**0.005**	**−0.45**	**0.045**	**−0.76**	**0.000**
*DGAT2*	**−0.44**	**0.004**		**−0.53**	**0.015**	−0.38	0.096	−0.10	0.690	**−0.50**	**0.025**
**Glucose metabolism genes**											
*IRS1*	−0.19	0.235		−0.05	0.849	−0.28	0.236	−0.10	0.681	−0.08	0.738
*GLUT4*	−0.30	0.061		−0.43	0.061	−0.30	0.194	−0.10	0.664	**−0.51**	**0.022**
*AKT*	−0.28	0.081		−0.33	0.153	−0.23	0.321	−0.14	0.555	−0.30	0.205
*TBC1D4*	**0.50**	**0.001**		−0.03	0.890	0.01	0.982	−0.03	0.904	00	1.000
**Genes in mitochondrial energy metabolism**											
*CPT1A*	−0.01	0.951		−0.32	0.166	0.17	0.475	−0.12	0.614	0.07	0.761
*CPT1B*	**−0.54**	**0.000**		**−0.60**	**0.005**	−0.22	0.344	**−0.62**	**0.004**	**−0.72**	**0.000**
**Adipogenic genes**											
*PPARG*	**−0.43**	**0.005**		−0.44	0.051	−0.43	0.056	−0.23	0.330	**−0.47**	**0.037**
*CEBPA*	**−0.50**	**0.001**		**−0.73**	**0.000**	−0.36	0.121	−0.34	0.147	**−0.73**	**0.000**

Bold values indicate statistical significance (*p* < 0.05)

A complete list of gene names can be found in Supplementary Table 3

*ND* non-diabetic

Moreover, *FKBP5* gene expression in SAT negatively correlated with expression levels of genes corresponding to key proteins involved in the lipolytic machinery. These included *ATGL* (*r* = −0.50), *MGLL* (*r* = −0.51), *PLIN3* (*r* = −0.47) and *PLIN4* (*r* = −0.43) (*p* < 0.01 for all). In contrast, *FKBP5* gene expression levels positively correlated with *PDE3B* (*r* = 0.60, *p* < 0.001) (Table 3).

*FKBP5* gene expression was also found to negatively correlate with the lipogenic genes *DGAT1* (*r* = −0.62, *p* < 0.001) and *DGAT2* (*r* = −0.44, *p* < 0.01) and the adipogenic genes *PPARG* and *CEBPA* (*r* = −0.43, −0.50, respectively; *p* < 0.01 for both) (Table 3).

In relation to genes involved in glucose metabolism, *FKBP5* gene expression was found to positively correlate with the gene expression levels of *TBC1D4* (*r* = 0.50, *p* < 0.01). There were also tendencies toward negative correlations between gene expression levels of *FKBP5* with *GLUT4* (*r* = −0.30, *p* = 0.061) and *Akt* (*r* = −0.28, *p* = 0.081). However, *FKBP5* gene expression levels did not correlate with basal or insulin-stimulated glucose uptake (Supplementary Table 1, Supplementary Figure 1).

### FKBP51 expression levels during differentiation of preadipocytes into adipocytes ex vivo

**Fig. 2:**
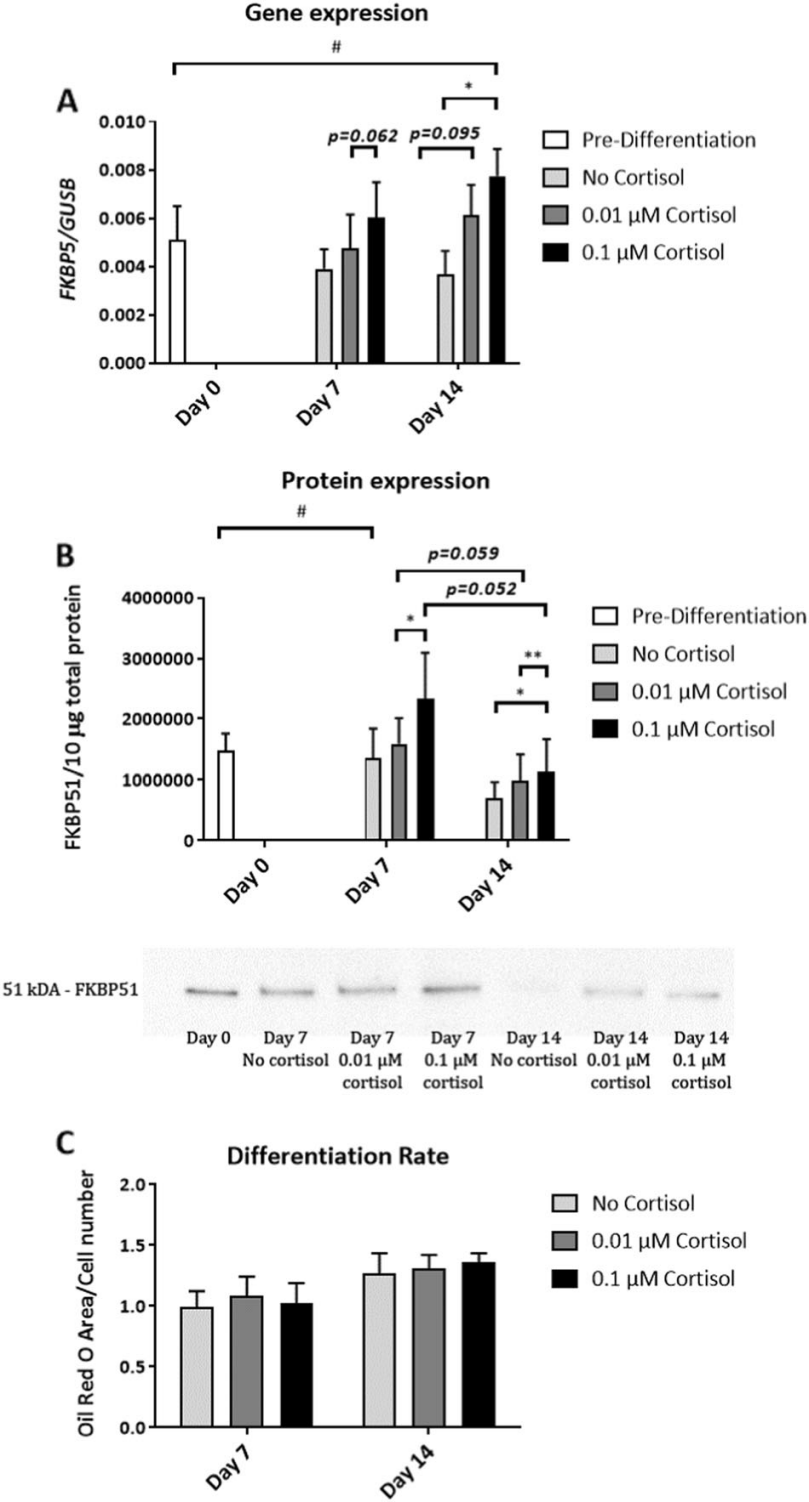
*FKBP5* expression during human preadipocyte differentiation into adipocyte. FKBP51 gene **a** and protein **b** expression levels in human preadipocytes before starting differentiation (pre-differentiation) and at days 7 and 14 after inducing differentiation into adipocytes without or with cortisol (0.01 and 0.1 µM) in the differentiation media (*n* = 5). Differentiation rate at days 7 and 14 of differentiation (*n* = 3), measured by image quantification of adipocyte lipids stained with Oil Red O, and normalised by cell number **c**. ^#^*p* < 0.05, between different days; *p < 0.05, **p < 0.01 between different treatments. Data were log(*x* + 1) transformed

Preadipocytes that differentiated into adipocytes in media with 0.1 µM cortisol for 14 days had about 2-fold higher gene expression levels of *FKBP5* compared to preadipocytes that differentiated in media with no cortisol during the same timeframe (*p* < 0.05, Fig. 2a).

At the protein level, cortisol treatment was shown to elicit a dose-response in FKBP51 expression. On day 7, preadipocytes differentiated with 0.1 µM cortisol showed 46% higher FKBP51 expression levels than those differentiated with 0.01 µM cortisol (*p* < 0.05, Fig. 2b). On day 14, preadipocytes differentiated with 0.1 µM cortisol showed 15% (*p* < 0.01) and 61% (*p* < 0.05) higher FKBP51 expression levels than those differentiated with 0.01 µM and no cortisol, respectively (Fig. 2b).

Moreover, preadipocytes differentiated in the presence of 0.01 and 0.1 µM cortisol showed a reduction tendency in FKBP51 protein expression levels from day 7 to 14 of differentiation of 38% (*p* = 0.059) and 51% (*p* = 0.052), respectively (Fig. 2b).

Varying concentrations of cortisol (0, 0.01 and 0.1 µM) did not affect the differentiation rate of preadipocytes into adipocytes at day 7 or day 14 (Fig. 2c, Supplementary Figure 2).

**Fig. 3:**
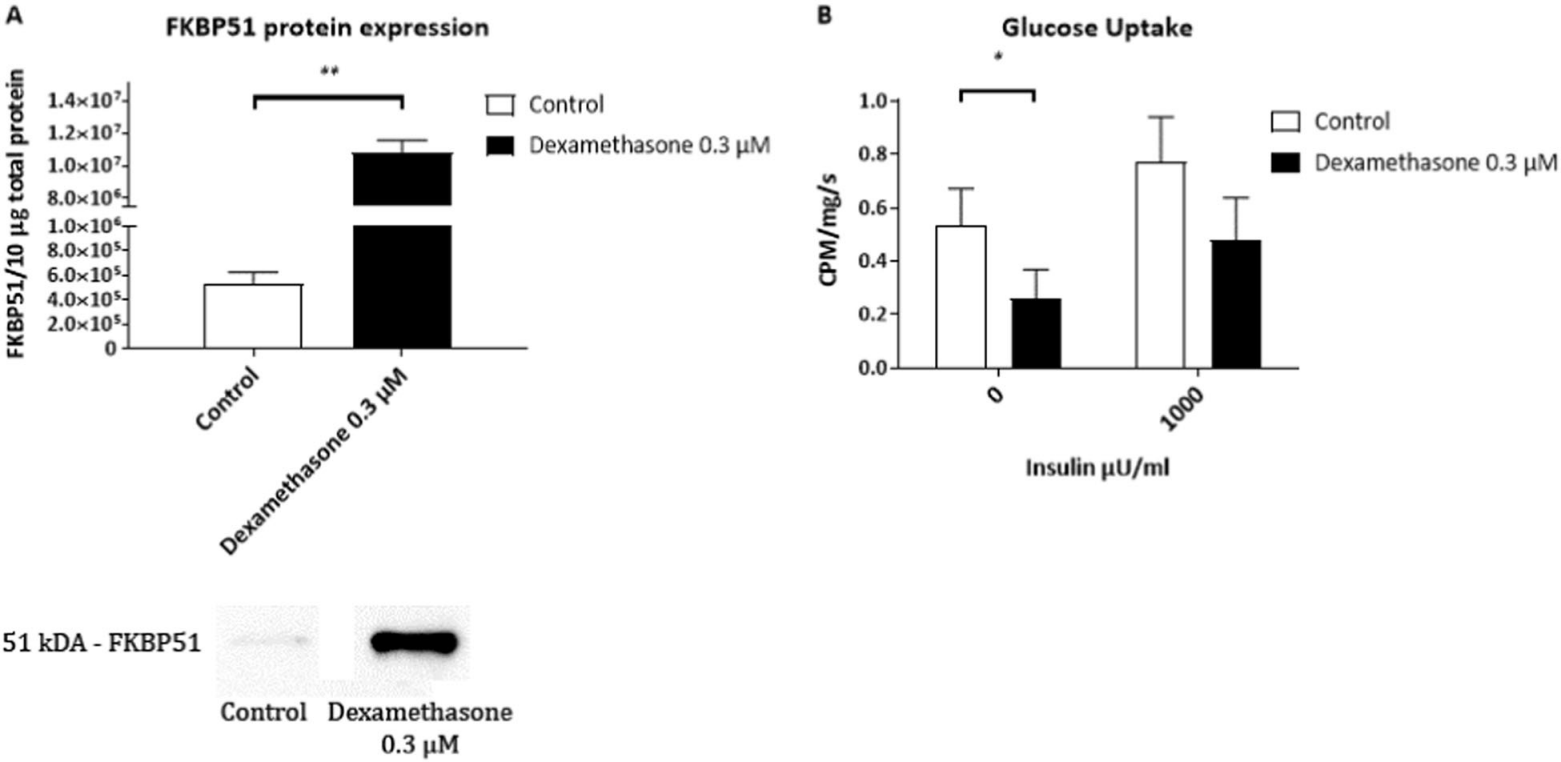
FKBP51 protein expression levels were 20-fold elevated in preadipocytes differentiated into adipocytes ex vivo following 24 h incubation with dexamethasone (0.3 µM) (*n* = 4) **a**. Incubation for 24 h with dexamethasone reduced basal glucose uptake in preadipocytes differentiated into adipocytes ex vivo by 51% (*n* = 4) **b**. Data were log(*x* + 1) transformed

Addition of dexamethasone for 24 h on day 16 of differentiation, increased FKBP51 gene and protein expression levels by 54-fold (*p* = 0.083, not shown) and 20-fold (*p* < 0.05, Fig. 3a), respectively.

Moreover, 24 h treatment with dexamethasone reduced the basal glucose uptake rate by 27% (*p* < 0.05) and showed a tendency to reduce the insulin-stimulated glucose uptake rate by 29% in ex vivo differentiated adipocytes (Fig. 3b).

### SAFit1 can prevent dexamethasone-induced impairment of glucose uptake in isolated primary adipocytes

**Fig. 4:**
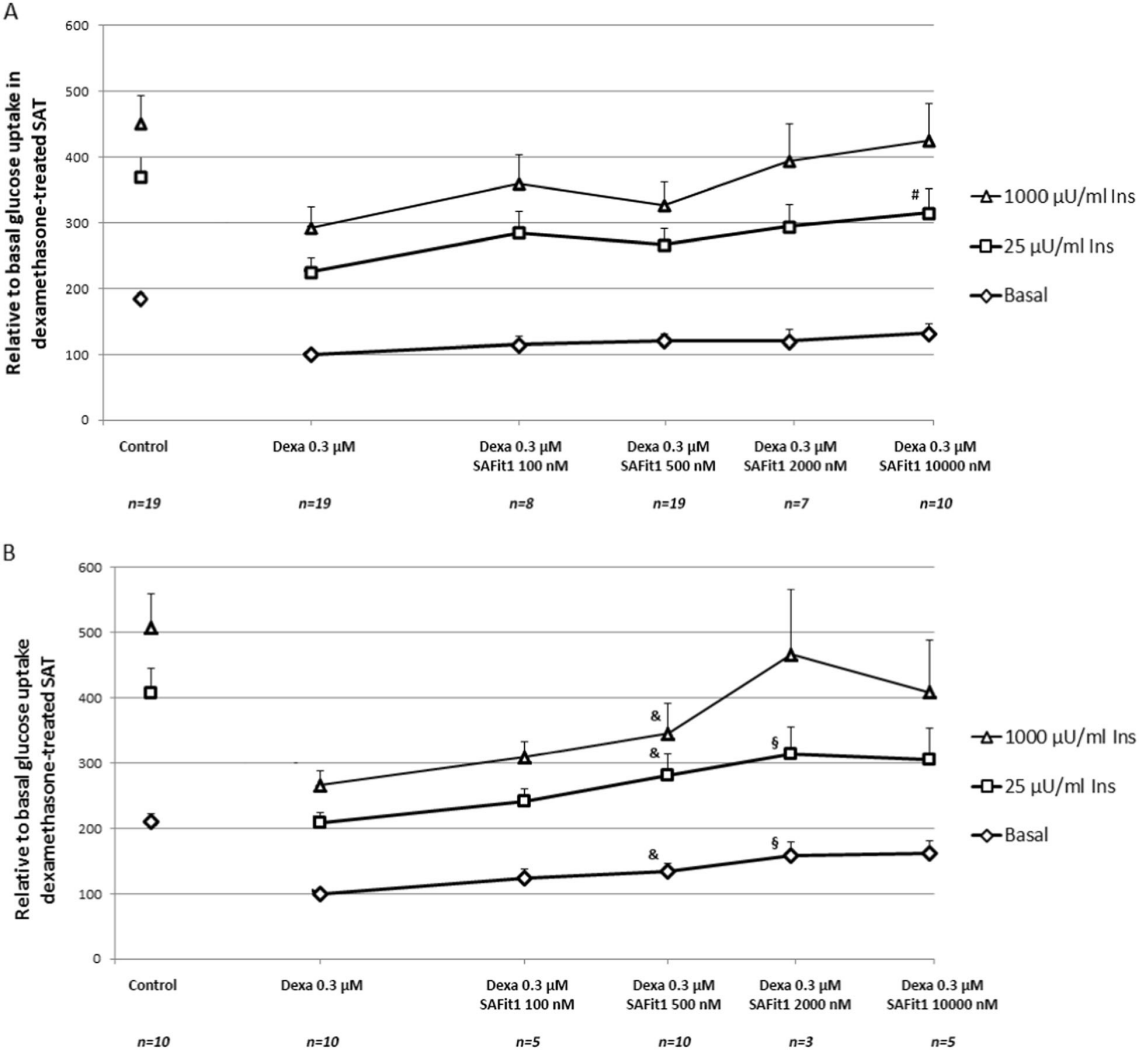
SAFit1 dose-response curve for adipocyte glucose uptake obtained from adipose tissue treated for 24 h with dexamethasone (0.3 µM) and with different concentrations of SAFit1 (100–10,000 nM) (*n* = 7–19) **a** and SAFit1 dose-response curve for adipocyte glucose uptake with higher percent inhibitory effect of dexamethasone on glucose uptake (50th percentile and above, *n* = 3–10), compared with control (no dexamethasone treatment), after 24 h of incubation **b**. For reference, the basal glucose uptake in all subjects was 38.0 femtoliter/cell/s (control). Values are percentage relative to basal dexamethasone. Dexa Dexamethasone, SAT subcutaneous adipose tissue. ^&^*p* < 0.05, Dexa + SAFit1 (500 nM) treated vs Dexa treated; ^§^*p* < 0.05, Dexa + SAFit1 (2000 nM) treated vs Dexa treated; ^#^*p* < 0.05, Dexa + SAFit1 (10,000 nM) treated vs Dexa treated

Incubation of SAT for 24 h with dexamethasone reduced basal, 25 and 1000 µU/ml insulin-stimulated glucose uptake by 42%, 37% and 32%, respectively (*p* < 0.001 for all) in isolated primary adipocytes (Fig. 4a). Overall, co-incubation with SAFit1 showed a dose-dependent trend to prevent impairment of basal and insulin-stimulated glucose uptake (Fig. 4a).

When dividing the individuals by the efficacy of dexamethasone treatment to inhibit glucose uptake, the responders (50th percentile and above) displayed a dose-dependent rescue by SAFit1 to at least partly normalise glucose uptake (Fig. 4b, Supplementary Table 4). See Supplementary Results for additional details on SAFit1 effects on adipocyte glucose uptake.

### Effects of SAFit1 treatment on expression levels of genes regulated by GR

As expected, treatment of SAT for 24 h with dexamethasone upregulated several genes known to be regulated by the GR-complex, including *FKBP5* (14-fold, *p* < 0.01)*, CNR1, GILZ, LPL* (230-, 5- and 1.26-fold, respectively; *p* < 0.001) and *PPARG* (1.22-fold, *p* < 0.05) (data not shown). Dexamethasone was also shown to downregulate the genes *GR, IL-6* (1.4- and 4-fold, respectively; *p* < 0.001), *HSP90AA1* (1.4-fold, *p* < 0.01) and *IRS-1* (1.2-fold, *p* < 0.05) (data not shown). Co-incubation with SAFit1 had no effect on these gene expression levels.

## Discussion

In this study, we expanded on our previous findings [12] by using a larger and more diverse cohort that includes non-diabetic and T2D subjects that were very well matched for sex, age and BMI. We show that *FKBP5* gene expression has strong links to insulin resistance, and that it displays a tendency of elevation in T2D subjects. We show that this association is mostly predicted by glucose AUC during OGTT data, with the strongest contributions to the association being observed in non-diabetic obese subjects. This may link into previous findings of the rs1360780 polymorphism of the *FKBP5* gene being associated to attenuated weight reduction following bariatric surgery of obese patients [19]. We previously reported that in 24 h incubated SAT, *FKBP5* gene expression correlates positively to fasting insulin [12]. In the present study fresh SAT did not show correlation. These differences may be due to the different treatments of the SAT—incubated SAT vs fresh SAT and the different biopsy procedures—surgical vs needle biopsies [38] used in previous [12] and in this study, respectively. Moreover, we found that *FKBP5* gene expression in SAT negatively correlated with genes corresponding to proteins involved in lipolysis. However, only in obese subjects was *FKBP5* gene expression levels found to be associated with the lipolytic rate of isolated adipocytes. The contribution of SVF, which contains several cell types, including blood cells, endothelial cells, adipose precursor cells, macrophages and fibroblasts, could potentially explain the absence of a correlation between *FKBP5* gene expression in SAT and lipolysis in isolated adipocytes.

*FKBP5* gene expression levels did not correlate with BMI, waist circumference or waist-hip ratio (WHR). This suggests that other obesity-associated mechanisms or states could be at play in a potential role of FKBP51 in lipid metabolism.

We found that *FKBP5* gene expression correlates negatively with two of the principal genes regulating adipogenesis, i.e. *PPARG* and *CEBPA* [39], suggesting that FKBP51 might be involved in adipocyte differentiation. Furthermore, recent studies have demonstrated that FKBP51 regulates PPARG activity, and therefore adipogenesis via the GR through an Akt-p38 kinase pathway [24]. It has also been demonstrated that the knockdown or knockout of the *FKBP5* gene in 3T3-L1 cells [24] or mouse embryonic fibroblasts [23, 24], respectively, has a strong anti-adipogenic impact in cells. It is, however, difficult to pinpoint the exact causality of *FKBP5* expression, or lack thereof, on adipogenesis. Our results seem to suggest that although FKBP51 levels tend to positively correlate with cortisol concentrations; these varying FKBP51 levels do not seem to affect the differentiation rate in human preadipocytes. However, with these experiments we do not exclude a role of FKBP51 on adipocyte differentiation, since even in cells differentiated in absence of cortisol there were basal levels of FKBP51 protein that could be sufficient to induce differentiation of preadipocytes into adipocytes. Further studies with complete knock-out of FKBP51 are warranted to explore the role of FKBP51 in human adipocyte differentiation. Furthermore, our data indicate that FKBP51 protein expression levels progressively decrease during differentiation. A reduction in FKBP51 levels during differentiation implies that FKBP51 may have a greater role in the earlier stages of adipogenesis rather than in later stages. This is in accordance with the reported roles of CEPBA and PPARG in the early stages of the differentiation process [40].

In line with previous findings [12], we showed that the synthetic glucocorticoid dexamethasone elevated FKBP51 gene and protein expression levels in human adipose tissue. We also found that dexamethasone had a similar effect on FKBP51 expression levels in adipocytes differentiated ex vivo. In addition, the effects of dexamethasone on inhibiting glucose uptake in primary adipocytes isolated from incubated SAT were also observed in adipocytes differentiated ex vivo. Addition of SAFit1 together with dexamethasone was shown to prevent the dexamethasone-induced impairment of glucose uptake of SAT incubated with dexamethasone alone. We found that this prevention by SAFit1 was dependent on the efficacy of dexamethasone treatment to inhibit glucose uptake. In short-term incubation conditions with SAFit1 (data not shown), no recovery of dexamethasone’s inhibitory effects on glucose uptake were observed. This suggests that the mechanism is unlikely to be mediated by the rapid phosphorylation events in the insulin signalling cascade. Furthermore, the effects of SAFit1 were similar in basal (no insulin) and insulin-stimulated conditions and there was no apparent shift in the insulin concentration-response curve, thus suggesting no effect on insulin signalling.

To better understand how FKBP51 may be involved in mediating glucose uptake, we examined the effects of dexamethasone/SAFit1 incubation on gene expression levels of genes downstream of the GR in SAT. We found that dexamethasone had significant effects on several GR-regulated genes, including GR expression itself and *FKBP5*. However, co-incubation of dexamethasone and SAFit1 had no additional effects. This may imply that SAFit1 does not interfere with the FKBP51–GR interaction. Furthermore, it may suggest that the mechanism by which SAFit1 prevents dexamethasone’s effects on glucose uptake, are not mediated via genetic downstream pathways.

An involvement of FKBP51 in Akt-pathways and its direct interaction with Akt/PKB has previously been demonstrated [20, 24, 41–43]. The FKBP51 protein consists of the two FKBP-type (FK) domains, FK1, FK2 and a tetratricopeptide repeat (TPR) domain [44]. The FK1 and FK2 domains interact with Akt whereas the TPR domain interacts with elements of the GR, where it regulates glucocorticoid affinity and downstream gene regulation of the GR complex [44]. Deletion of residues corresponding to the two Akt-associated domains has been shown to abolish Akt interaction [20]. A SAFit1 analogue was shown reorganise the Akt2–AS160 complex in muscle tissue of mice [45], but ligands binding to the FK506/SAFit1-binding pocket in the FK1 domain of FKBP51 did not affect the FKBP51–Akt interaction [46].

A recent study found that FKBP51-knockout mice had improved insulin-mediated glucose uptake in skeletal muscle compared to wild type mice [45]. In line with our findings, the study found that FKBP51 regulates Akt–AS160 phosphorylation. In contrast to our findings, glucose metabolism did not differ in white adipose tissue of the FKBP51-knockout mice compared to wild type mice. Based on these findings, it would be of interest to further investigate a possible interplay of FKBP51–Akt–AS160 and to evaluate FKBP51 inhibition in human skeletal muscle.

This study has several limitations. First it is limited by its exploratory nature, and the associations between *FKBP5* levels and the expression levels of other genes do not demonstrate causality. Future studies should closer examine FKBP51 in relation to the other gene products highlighted here. Second, an examination of FKBP51 in SAT alone may not convey much of a systemic role of FKBP51. Therefore, it would be of interest to dissect FKBP51's role in other metabolically active tissues; in particular skeletal muscle tissue and liver which quantitatively account for much of insulin-mediated glucose metabolism. Third, it is an ex vivo study, therefore the impact of the needle biopsy on the adipose tissue gene expression and adipocyte metabolism is unknown. Fourth, the study is further limited by the unknown exact mechanism of action of SAFit1. We cannot completely rule out effects of SAFit1 on phosphorylation in the insulin signalling cascade. In this paper, we have further underscored the role of FKBP51 in metabolism and, specifically, its link to insulin resistance and T2D in SAT. Previously published SNP data of the *FKBP5* gene region have showed several SNPs being associated to T2D and related phenotypes [12]. In addition, the gene variant rs1360780 was recently shown to be associated with WHR [47, 48]. These findings give additional support to an association between FKBP51 and T2D-related phenotypes.

Moreover, we demonstrate a potential direct role of FKBP51 in the glucose turnover machinery. This was demonstrated by the use of the FKBP51-specific inhibitor SAFit1 to prevent dexamethasone-induced insulin resistance in human adipose tissue.

Having established a glucose uptake assay in adipocytes differentiated ex vivo, it would be of interest to completely knock out the expression of the *FKBP5* gene in human preadipocytes. This would help clarify the involvement of FKBP51 in adipogenesis and its role in the glucose uptake machinery that is impaired by dexamethasone in SAT. Regulation of FKBP51 by different levels of cortisol during differentiation does not affect the differentiation rate. An *FKBP5* knockout model may clarify why. The purported protein–protein interaction between FKBP51, Akt and possibly AS160 should also be further investigated.

In conclusion, we have found that *FKBP5* expression in human subcutaneous adipose tissue is associated with T2D traits and markers of insulin resistance. It can also partly mediate effects of glucocorticoids to impair glucose utilisation, and this may partly be prevented by a selective FKBP51 inhibitor. Furthermore, *FKBP5* gene expression is associated with genes involved in lipid metabolism and adipogenesis, thus further supporting the involvement of FKBP51 in metabolic regulation.

## Electronic supplementary material

Supplementary Data

Supplementary Figure1

Supplementary Figure2

Supplementary Figure3

Supplementary Figure4

## Abbreviations

AUC: area under the curve
DAPI: diamidino-2-phenylindole
DM: differentiation medium
DMEM: Dulbecco’s modified Eagle medium
FBS: fetal bovine serum
FK: FKBP-type
FKBP51: FK506 binding protein 51
FKBP52: FK506 binding protein 52
GR: glucocorticoid receptor
HPA-axis: hypothalamic–pituitary–adrenal axis
Hsp90: heat shock protein 90
Matsuda: Matsuda insulin sensitivity index
OGTT: oral glucose tolerance test
PEST: penicillin–streptomycin
PKB/Akt: protein kinase B
SAT: subcutaneous adipose tissue
SVF: stromal vascular fraction
TPR: tetratricopeptide repeat
T2D: type 2 diabetes
