# Supplementary material for: FKBP5 expression in human adipose tissue: potential role in glucose and lipid metabolism, adipogenesis and type 2 diabetes
Source: Endocrine. 2018 Jul 21;62(1):116–28. doi: 10.1007/s12020-018-1674-5 (PMC6153563; doi:10.1007/s12020-018-1674-5)
Supplement: Supplementary file 1 — Supplementary Data [file 12020_2018_1674_MOESM1_ESM.docx]

**FKBP5 expression in human adipose tissue: Potential role in glucose and lipid metabolism, adipogenesis and type 2 diabetes**

Cherno O Sidibeh^1^, Maria J Pereira^1^, Xesus M Abalo^1^, Gretha Boersma^1^, Stanko Skrtic^2,3^, Per Lundkvist^1^, Petros Katsogiannos^1^, Felix Hausch^4^, Casimiro Castillejo-López^1^, Jan W Eriksson^1^

^1^Department of Medical Sciences, Uppsala University, Uppsala, Sweden

^2^AstraZeneca R&D, Mölndal, Sweden

^3^Institute of Medicine, Sahlgrenska Academy, University of Gothenburg, Gothenburg, Sweden

^4^Institute of Organic Chemistry and Biochemistry, Technical University Darmstadt, Darmstadt, Germany

*Corresponding author contact info***:**

Jan W Eriksson

Department of Medical Sciences, Uppsala University, 751 85 Uppsala, Sweden

Phone: +46 186114419

e-mail: [jan.eriksson@medsci.uu.se](mailto:jan.eriksson@medsci.uu.se)

**Supplementary Appendix**

**Supplementary Materials and Methods**

## Glucose uptake

Primary adipocytes were isolated from subcutaneous adipose tissue (SAT) with collagenase (Sigma-Aldrich, St. Louis, Missouri, USA) and washed in glucose-free Krebs-Ringer-Hepes (KRH), supplemented with 4% bovine serum albumin (BSA), 150 nM adenosine, pH 7.4. Adipocytes were diluted ten times in KRH medium and were stimulated or not with insulin (1000 µU/ml) for 15 min in a shaking water bath at 37 °C. Thereafter, D-[U-14C] glucose (0.26 mCi/L, 0.86 µM; NEC042V250UC, PerkinElmer, Waltham, Massachusetts, USA) was added and the accumulation of glucose followed for 45 min. The reaction was stopped by the transfer of the cell suspension into ice-cold tubes with silicon oil (SERVA Electrophoresis GmbH, Heidelberg, Germany) and the adipocyte-associated radioactivity was measured in a beta-counter. Glucose uptake was determined by the rate of transmembrane glucose transport and calculated as previously described [1].

## Lipolysis

Primary adipocytes were isolated from SAT with collagenase (Sigma-Aldrich) and washed with Hank´s medium 199 (Gibco, Paisley, UK) supplemented with 4% BSA (Sigma), 150 nM adenosine (Sigma), pH 7.4. Adipocytes were diluted to a lipocrit of 2-3% and the cell suspension was incubated with or without isoproterenol (0.5 µM, Sigma) and with or without insulin (100 µU/mL, Actrapid, Novo Nordisk, Bagsvaerd, Denmark) in a gently shaking water-bath at 37 °C for 2 h. Glycerol released into the media was measured by colorimetric absorbance at 540 nm with Free Glycerol Reagent (Sigma-Aldrich). Cellular lipids were extracted [2] and adipocyte size and cell number was measured as previously described [3]. The glycerol released into the media was normalized per cell number and calculated relative to basal control in each experiment.

## mRNA levels assessment

mRNA expression levels of *FKBP5*, other glucocorticoid-regulated genes and genes involved in adipogenesis and glucose, lipid and energy metabolism, in SAT from Cohort 1, were measured by RNaseq at Exiqon A/S, Vedbaek, Denmark, as previously described [4].

For real-time PCR assays, RNA was isolated from incubated adipose tissue from Cohort 2 subjects or adipocytes in culture using the RNeasy Lipid Tissue Mini Kit (Qiagen, Hilden, Germany) according to manufacturer’s protocol. Purified RNA was quantified using a NanoDrop ND-1000 spectrophotometer (NanoDrop Technologies, Wilmington, DE, USA). This was followed by conversion of RNA to cDNA using a high-capacity cDNA reverse transcription kit (Applied Biosystems, Foster City, CA, USA) and relative quantification using TaqMan probes targeting the genes *FKBP5*, *Akt, CNR1, GILZ, GLUT4, GR, HSP90, IL6, IRS1, LEP, LPL* and *PPARG* mRNA (Supplementary Table 2) was performed. All reagents for real-time PCR were purchased from Applied Biosystems and used according to manufacturer's protocol. The relative quantification of gene expression was analysed using the QuantStudio 3 Real-Time PCR System (Applied Biosystems). A standard curve of pooled cDNA was used to determine the concentration of the target gene. Gene expression levels were normalized to the housekeeping gene *GUSB*.

## Stromal vascular fraction (SVF) assessment

To assess the involvement of FKBP51 in human adipogenesis, the SVF, that contains preadipocytes, was isolated from human SAT. Fresh SAT from Cohort 2 was treated with collagenase, in order to separate the SVF cells from the mature adipocytes, as previously described [4,5]. The SVF was then cultured in preadipocyte medium, DMEM: Nutrient Mixture F-12 (DMEM/F12, 10% FBS, 1% PEST, 0.4% Gentamicin, Thermo Scientific, Waltham, Massachusetts, USA; 4.25 ng/ml human basic fibroblast growth factor, Sigma) until reaching a confluency of 70-80%. Cells were expanded until ~1 000 000 cells and this usually required no more than 2 passages. Then, preadipocytes were transferred to 24-well plates at a density of 15 000 cells/cm^2^. Upon reaching 100% confluence, cells were differentiated as previously described [6,7]. In brief, differentiation was induced using differentiation medium (DM, DMEM/F12, 1% PEST, 100 nM Insulin, 17 µM panthothenate, 33 µM biotin, 0.1 µM cortisol, 1 µM rosiglitazone, 10 µg/ml transferrin, 2 nM triiodothyronine) supplemented with 250 µM 3-isobutyl-1-methylxanthine for the first 5 days only. Preadipocytes were maintained in DM for a total of 14 days, with medium being replenished twice per week. Samples for gene and protein expression were collected on days 0, 7 and 14 (*n=*5). In addition, Oil Red O staining and subsequent imaging of differentiating cells was performed on days 7 and 14 of differentiation (*n=*3).

FKBP51 expression is strongly regulated by cortisol levels [8]. Glucocorticoids, such as cortisol, are important for the differentiation of preadipocytes into adipocytes [9,10]. Therefore, preadipocytes from some subjects (*n=*5) were also differentiated in culture conditions with no, low or standard cortisol levels (0, 0.01 and 0.1 µM, respectively).

## Imaging and quantification of adipocyte differentiation

The lipids of the adipocytes undergoing *ex-vivo* differentiation were stained with Oil Red O solution to quantify lipid accumulation and thereby the rate of differentiation. Media was removed and wells were washed twice with PBS. 4% formaldehyde was added for 30 minutes at room temperature. Formaldehyde was removed and the cells were washed twice with ddH_2_O. The lipids of the adipocytes were then stained with 60% Oil Red O solution for 30 minutes. Oil Red O solution was removed and cells were again washed twice with ddH_2_O. The nuclei of the adipocytes were then stained with 300 nM 4',6-diamidino-2-phenylindole (DAPI) for 10 minutes, washed twice with ddH_2_O and then stored in 1 ml of water per well.

Adipocyte differentiation was measured by image quantification of cells stained with Oil Red O [11]. The EVOS FL Auto-fluorescent microscope (Thermo Scientific, Waltham, Massachusetts, USA) was used to image the lipids on the transmitted channel and the EVOS® DAPI Light Cube to capture the nuclei. Pixels per image were normalized with the number of cell nuclei using Hoechst® 33342 (1.8 µM). Random images were automatically recorded from duplicate cultures and analysed at batch mode using ImageJ 1.50i (Rasband, 1997). An average of 1800 cells was analysed for each cell culture.

The lipids of the adipocytes undergoing *ex-vivo* differentiation were stained with Oil Red O solution to quantify lipid accumulation and thereby the rate of differentiation. Media was removed and wells were washed twice with PBS. 4% formaldehyde was added for 30 minutes at room temperature. Formaldehyde was removed and the cells were washed twice with ddH_2_O. The lipids of the adipocytes were then stained with 60% Oil Red O solution for 30 minutes. Oil Red O solution was removed and cells were again washed twice with ddH_2_O. The nuclei of the adipocytes were then stained with 300 nM 4',6-diamidino-2-phenylindole (DAPI) for 10 minutes, washed twice with ddH_2_O and then stored in 1 ml of water per well.

Adipocyte differentiation was measured by image quantification of cells stained with Oil Red O [11]. The EVOS FL Auto-fluorescent microscope (Thermo Scientific, Waltham, Massachusetts, USA) was used to image the lipids on the transmitted channel and the EVOS® DAPI Light Cube to capture the nuclei. Pixels per image were normalized with the number of cell nuclei using Hoechst® 33342 (1.8 µM). Random images were automatically recorded from duplicate cultures and analysed at batch mode using ImageJ 1.50i (Rasband, 1997). An average of 1800 cells was analysed for each cell culture.

**Supplementary Results**

***Association between FKBP5 gene expression levels in SAT and metabolic parameters***

*FKBP5* gene expression levels positively correlated with markers of insulin resistance, including the glucose area under the curve (AUC) during OGTT (*r=0.33, p<0.05*), fasting glucose (*r=0.47, p<0.01*), HOMA-IR (*r=0.34, p<0.05*) and fasting insulin resistance index (FIRI, *r=0.34, p<0.05*). *FKBP5* gene expression also correlated negatively with Matsuda insulin sensitivity index (Matsuda, *r=-0.34, p<0.05*), Gutt insulin sensitivity index (Gutt, *r=-0.32, p<0.05*) and Quantitative Insulin Sensitivity Check index (QUICKI, *r=-0.33, p<0.05*). An association was also found between *FKBP5* gene expression and systolic blood pressure (SBP) (*r=0.32, p<0.05*). After inclusion of glucose AUC during OGTT, HOMA-IR, Matsuda and SBP in a multivariate regression analysis; glucose AUC during OGTT (standard β coefficient=0.55, *p<0.05*; model: *r^2^=0.27*) remained the only significant predictor of *FKBP5* gene expression in SAT (Table 2). When subdividing the subjects by T2D and obesity status, the *FKBP5* gene expression was associated with glucose AUC during OGTT only in the non-diabetic (*r=0.41, p=0.076*) and obese (*r=0.49, p<0.05*) subjects (Supplementary Table 1). *FKBP5* gene expression did not correlate with BMI, WHR and fasting insulin (data not shown).

When the Cohort 1 subjects were subdivided in terms of T2D and obesity status and sex; glucose AUC during OGTT remained a significant predictor of *FKBP5* gene expression only in obese subjects (*r=0.49, p<0.05*) (Supplementary Table 1).

***FKBP5 expression levels during differentiation of preadipocytes into adipocytes ex vivo***

Preadipocytes that differentiated into adipocytes in media with 0.1 µM cortisol for 14 days had about 2-fold higher gene expression levels of *FKBP5* compared to preadipocytes that differentiated in media with no cortisol during the same timeframe (*p<0.05*, Figure 2A).

At the protein level, cortisol treatment was shown to elicit a dose-response in FKBP51 expression. On day 7, preadipocytes differentiated with 0.1 µM cortisol showed 46% higher FKBP51 expression levels than those differentiated with 0.01 µM cortisol (*p<0.*05, Figure 2B). On day 14, preadipocytes differentiated with 0.1 µM cortisol showed 15% (*p<0.*01) and 61% (*p<0.*05) higher FKBP51 expression levels than those differentiated with 0.01 µM and no cortisol, respectively (Figure 2B).

Moreover, preadipocytes differentiated in the presence of 0.01 and 0.1 µM cortisol showed a reduction tendency in FKBP51 expression levels from day 7 to 14 of differentiation of 38% (*p=0.059)* and 51% (*p=0.052*), respectively (Figure 2B).

Preadipocytes that differentiated into adipocytes in media in the absence of cortisol had similar *FKBP5* gene expression levels at day 7 and 14, but their protein levels show a numerical reducing trend at day 14, compared with day 7 (not significant, Figure 2A and B).

Varying concentrations of cortisol (0, 0.01 and 0.1 µM) for the *ex vivo* differentiation of preadipocytes did not affect the differentiation rate at day 7 or day 14 (Figure 2C, Supplementary Figure 2).

Addition of dexamethasone for 24 h on day 16 of differentiation, increased FKBP51 gene and protein expression levels by 54-fold (*p=0.083,* not shown) and 20-fold (*p<0.05,* Figure 3A), respectively.

Moreover, 24 h treatment with dexamethasone reduced the basal glucose uptake rate by 27% (*p<0.05*) and showed a tendency to reduce the insulin-stimulated glucose uptake rate by 29% in *ex vivo* differentiated adipocytes (Figure 3B).

***SAFit1 can prevent dexamethasone-induced impairment of glucose uptake in adipocytes***

Incubation of SAT for 24 h with dexamethasone reduced basal, 25- and 1000 µU/ml insulin-stimulated glucose uptake by 42%, 37% and 32%, respectively (*p<0.001* for all) in isolated primary adipocytes (Figure 4A). Overall, co-incubation with SAFit1 showed a dose-dependent trend to prevent impairment of basal and insulin-stimulated glucose uptake (Figure 4A).

However, reduction in glucose uptake following dexamethasone treatment varied substantially between subjects and a range from about 5 to 57% reduction in 1000 µU/ml insulin-stimulated glucose uptake was observed. When dividing the individuals by the efficacy of dexamethasone treatment to inhibit glucose uptake, the responders (50^th^ percentile and above) displayed a dose-dependent rescue by SAFit1 to at least partly normalize glucose uptake (Figure 4B). In contrast, adipocyte recovery in glucose uptake following SAFit1 treatment was very low in subjects where the inhibitory effect of dexamethasone on glucose uptake was low (below 50^th^ percentile, data not shown). In fact, there was an inverse correlation between the percent inhibitory effect of dexamethasone on glucose uptake and the percent recovery with dexamethasone and SAFit1 combined treatment on 25- (*r=-0.45, p=0.054*) (Supplementary Figure 3A) and 1000 µU/ml insulin-stimulated glucose uptake (*r=-0.45, p=0.051*) (Supplementary Figure 3B).

Furthermore, incubation of SAT with SAFit1 alone, at various concentrations, did not affect the adipocyte glucose uptake (data not shown), suggesting that SAFit1’s effects on glucose uptake are dependent on the effect of dexamethasone.

**Supplementary Tables**

**Table 1 -** Association between *FKBP5* gene expression and metabolic parameters in Cohort 1 subjects

|  | | | | | | | | | | | | | | |
| --- | --- | --- | --- | --- | --- | --- | --- | --- | --- | --- | --- | --- | --- | --- |
|  | All | | ND | | T2D | | Male | | Female | | Non-obese | | Obese | |
|  | **r** | **p** | **r** | **p** | **r** | **p** | **r** | **p** | **r** | **p** | **r** | **p** | **r** | **p** |
| **Fasting insulin** | 0.22 | 0.167 | 0.35 | 0.133 | -0.04 | 0.860 | 0.42 | 0.064 | 0.22 | 0.356 | 0.30 | 0.201 | 0.31 | 0.185 |
| **AUC Glucose** | **0.33** | **0.039** | 0.41 | 0.076 | 0.24 | 0.310 | 0.32 | 0.171 | 0.11 | 0.650 | 0.19 | 0.413 | **0.49** | **0.028** |
| **HbA_1c_** | 0.27 | 0.096 | 0.21 | 0.386 | 0.12 | 0.63 | 0.20 | 0.388 | 0.32 | 0.170 | 0.01 | 0.977 | **0.59** | **0.007** |
| **HOMA-IR** | **0.34** | **0.033** | 0.34 | 0.143 | 0.25 | 0.292 | **0.37** | **0.112** | **0.34** | **0.148** | **0.23** | **0.339** | **0.59** | **0.007** |
| **FIRI** | **0.34** | **0.033** | 0.34 | 0.143 | 0.25 | 0.292 | 0.37 | 0.112 | 0.34 | 0.148 | 0.23 | 0.339 | **0.59** | **0.007** |
| **Matsuda** | **-0.34** | **0.034** | -0.40 | 0.084 | -0.11 | 0.631 | -0.41 | 0.077 | -0.30 | 0.195 | -0.28 | 0.225 | **-0.47** | **0.036** |
| **Gutt** | **-0.32** | **0.046** | -0.34 | 0.146 | 0.03 | 0.900 | -0.44 | 0.052 | -0.18 | 0.439 | -0.32 | 0.73 | **-0.42** | **0.069** |
| **QUICKI** | **-0.33** | **0.036** | -0.33 | 0.151 | -0.26 | 0.271 | -0.37 | 0.107 | -0.34 | 0.146 | -0.24 | 0.311 | **-0.57** | **0.009** |
| **HDL-cholesterol** | -0.07 | 0.648 | 0.05 | 0.823 | -0.19 | 0.421 | 0.34 | 0.146 | -0.36 | 0.119 | -0.04 | 0.869 | -0.09 | 0.71 |
| **LDL-cholesterol** | 0.04 | 0.800 | 0.20 | 0.403 | -0.08 | 0.724 | 0.07 | 0.779 | 0.07 | 0.781 | -0.03 | 0.902 | 0.00 | 0.990 |
| **Triglycerides** | 0.18 | 0.279 | 0.19 | 0.424 | 0.24 | 0.318 | 0.25 | 0.282 | 0.07 | 0.762 | 0.03 | 0.900 | 0.27 | 0.258 |
| **BMI** | 0.02 | 0.921 | 0.07 | 0.782 | -0.04 | 0.885 | 0.02 | 0.925 | 0.30 | 0.200 | 0.32 | 0.166 | 0.12 | 0.618 |
| **WHR** | 0.22 | 0.169 | 0.10 | 0.673 | 0.35 | 0.126 | -0.22 | 0.349 | 0.02 | 0.942 | 0.36 | 0.125 | 0.23 | 0.329 |
| **SC adipocyte diameter** | -0.03 | 0.847 | 0.09 | 0.715 | -0.10 | 0.663 | 0.04 | 0.882 | 0.34 | 0.142 | -0.02 | 0.947 | -0.03 | 0.910 |
| **SBP** | **0.32** | **0.044** | 0.28 | 0.227 | 0.25 | 0.282 | 0.42 | 0.067 | 0.17 | 0.47 | 0.36 | 0.12 | 0.40 | 0.081 |
| **DBP** | 0.26 | 0.101 | 0.00 | 0.995 | **0.60** | **0.005** | 0.00 | 0.990 | 0.20 | 0.400 | 0.29 | 0.211 | 0.32 | 0.18 |
| **Basal Glucose Uptake** | -0.01 | 0.972 | 0.22 | 0.359 | -0.29 | 0.229 | 0.24 | 0.333 | 0.07 | 0.782 | -0.03 | 0.892 | -0.01 | 0.985 |
| **25 µU/ml Insulin**  **Glucose Uptake** | 0.00 | 0.981 | 0.12 | 0.627 | -0.22 | 0.376 | 0.34 | 0.181 | 0.05 | 0.826 | 0.15 | 0.565 | -0.10 | 0.673 |
| **1000 µU/ml Insulin Glucose Uptake** | -0.12 | 0.477 | 0.12 | 0.613 | -0.36 | 0.128 | 0.19 | 0.433 | 0.01 | 0.975 | -0.07 | 0.786 | -0.16 | 0.490 |
| **Basal Lipolysis** | -0.14 | 0.405 | 0.06 | 0.816 | -0.40 | 0.078 | -0.20 | 0.402 | -0.08 | 0.738 | -0.34 | 0.139 | 0.06 | 0.801 |
| **0.5 µM Isoproterenol Lipolysis (Max)** | -0.25 | 0.114 | -0.22 | 0.342 | -0.32 | 0.166 | -0.36 | 0.123 | -0.13 | 0.587 | -0.01 | 0.965 | -0.34 | 0.148 |
| **% Max to Basal Lipolysis** | -0.17 | 0.308 | -0.40 | 0.082 | 0.05 | 0.826 | -0.14 | 0.556 | -0.16 | 0.510 | 0.23 | 0.33 | **-0.46** | **0.041** |

ND = Non-diabetic, AUC = Area under the curve;0 HbA_1c_ = Glycated haemoglobin; HOMA-IR = Homeostatic model assessment of insulin resistance; FIRI = Fasting insulin resistance index; Matsuda = Matsuda insulin sensitivity index; Gutt = Gutt insulin sensitivity index; QUICKI = Quantitative insulin sensitivity check index ; HDL = High density lipoprotein; LDL = Low density lipoprotein; BMI = Body mass index; WHR = Waist-hip ratio; SC = Subcutaneous; SBP = Systolic blood pressure; DBP = Diastolic blood pressure

**Table 2 –** Used Taqman probe target genes for real-time PCR and their corresponding assay ID at Applied Biosystems

| **Taqman probe target gene** | **Assay ID** |
| --- | --- |
| *FKBP5* | **Hs01561006_m1** |
| *Akt* | **Hs00178289_m1** |
| *CNR1* | **Hs01038522_s1** |
| *GILZ* | **Hs00608272_m1** |
| *GLUT4* | **Hs00168966_m1** |
| *GR* | **Hs00353740_m1** |
| *HSP90* | **Hs00743767_sH** |
| *IL6* | **Hs00985639_m1** |
| *IRS1* | **Hs00178563_m1** |
| *LEP* | **Hs00174877_m1** |
| *LPL* | **Hs00173425_m1** |
| *PPARG* | **Hs01115513_m1** |
| *GUSB* | **Hs00939627_m1** |

**Table 3 –** List of various metabolic gene symbols and names used to explore the association with FKBP5 gene expression in subcutaneous adipose tissue.

| **Gene symbol** | **Gene name** |
| --- | --- |
| **GR regulated genes** |  |
| *HSD11B1* | Hydroxysteroid 11-Beta Dehydrogenase 1 |
| *GR* | Glucocorticoid receptor |
| *HSP90AA1* | Heat Shock Protein 90 Alpha Family Class A Member 1 |
| *HSP90AB1* | Heat Shock Protein 90 Alpha Family Class B Member 1 |
| *HSP90B1* | Heat Shock Protein 90 Beta Family Member 1 |
| *CNR1* | Cannabinoid Receptor 1 |
| *GILZ* | TSC22 Domain Family Member 3 |
| *TIMP4* | TIMP Metallopeptidase Inhibitor 4 |
| **Genes of inflammatory cytokines** |  |
| *TNF* | Tumor Necrosis Factor |
| *IFNG* | Interferon Gamma |
| **Genes of adipokines** |  |
| *ADIPOQ* | Adiponectin |
| *LEP* | Leptin |
| **Lipid metabolism genes** |  |
| *PDE3B* | Phosphodiesterase 3B |
| *ATGL* | Adipose Triglyceride Lipase |
| *LIPE* | Hormone sensitive lipase |
| *PLIN1* | Perilipin 1  Perilipin 2 |
| *PLIN2* | Perilipin 2 |
| *PLIN3* | Perilipin 3 |
| *PLIN4* | Perilipin 4 |
| *LPL* | Lipoprotein lipase |
| *DGAT1* | Diacylglycerol acyltransferase 1 |
| *DGAT2* | Diacylglycerol acyltransferase 2 |
| **Glucose metabolism genes** |  |
| *IRS1* | Insulin receptor substrate 1 |
| *GLUT4* | Glucose transporter 4 |
| *AKT* | **AKT** Serine/Threonine Kinase 1 |
| *TBC1D4* | TBC1 Domain Family Member 4 |
| **Genes in mitochondrial energy metabolism** |  |
| *CPT1A* | Carnitine Palmitoyltransferase 1A |
| *CPT1B* | Carnitine Palmitoyltransferase 1B |
| **Adipogenic genes** |  |
| *PPARG* | Peroxisome proliferator activated receptor gamma |
| *CEBPA* | CCAAT/Enhancer Binding Protein Alpha |

**Table 4 –** Single subject data from adipocyte glucose uptake isolated from adipose tissue treated without (control) or with dexamethasone (0.3 µM) and different SAFit1 concentrations (nM) (high-responder to dexamethasone treatment).

|  | **Adipocyte glucose uptake (relative to basal-dexa treated)** | | | | | |
| --- | --- | --- | --- | --- | --- | --- |
|  | Control | Dexa 0.3 µM | | | | |
|  |  | SAFit1 (nM) | | | | |
|  |  | 0 | 100 | 500 | 2000 | 10000 |
| Basal | 228 | 100 | 78 | 82 | 188 | 176 |
| 25 µU/ml insulin | 650 | 267 | 198 | 375 | 375 | 460 |
| 1000 µU/ml insulin | 867 | 375 | 351 | 494 | 637 | 685 |

**Supplementary Figures**

**Supplementary Figure 1 –** Association between *FKBP5* gene expression in SAT and adipocyte glucose uptake and lipolysis in Cohort 1 subjects.

**
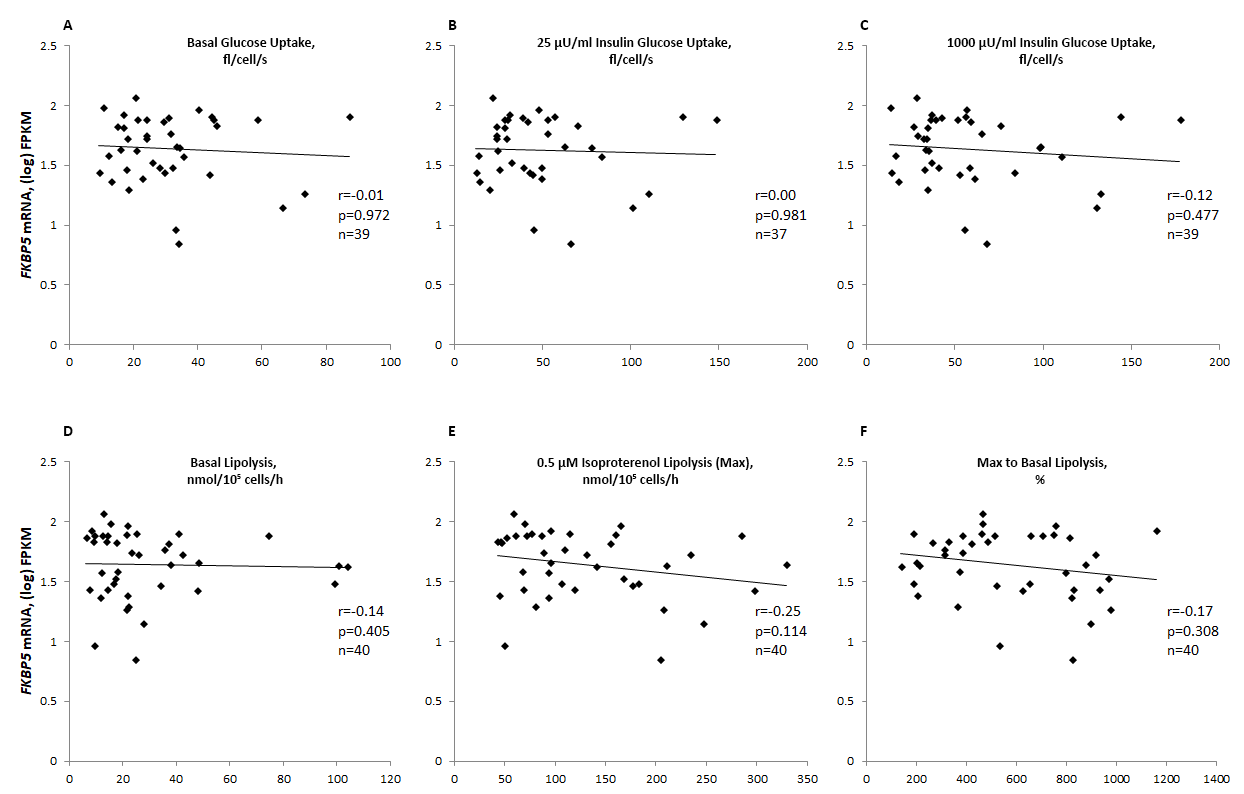
**

**Supplementary Figure 2 –** Images on day 14 of differentiation of adipocytes differentiated ex-vivo with 0 µM (A), 0.01 µM (B) and 0.1 µM (C) cortisol. Lipids are stained with Oil Red O (grey colour) and nuclei are stained with DAPI (blue colour).

**
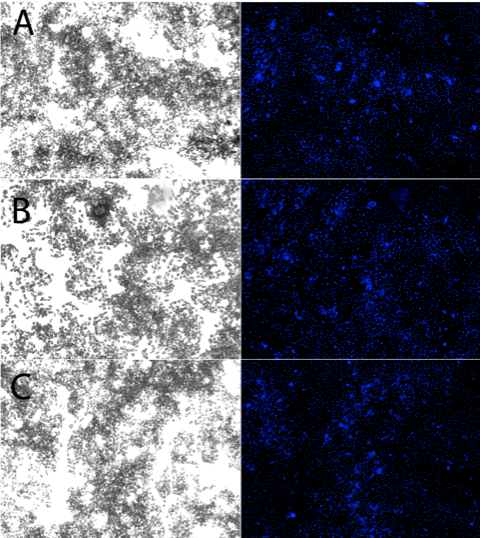
**

**Supplementary Figure 3**  – The percent inhibitory effect of dexamethasone (0.3 µM) on 25- (A) and 1000 µU/ml (B) insulin-stimulated glucose uptake on isolated adipocytes from SAT treated for 24 h with dexamethasone, negatively correlated with the percent recovery of dexamethasone (0.3 µM) and SAFit1 (500 nM) combined (*n=19)*.

**
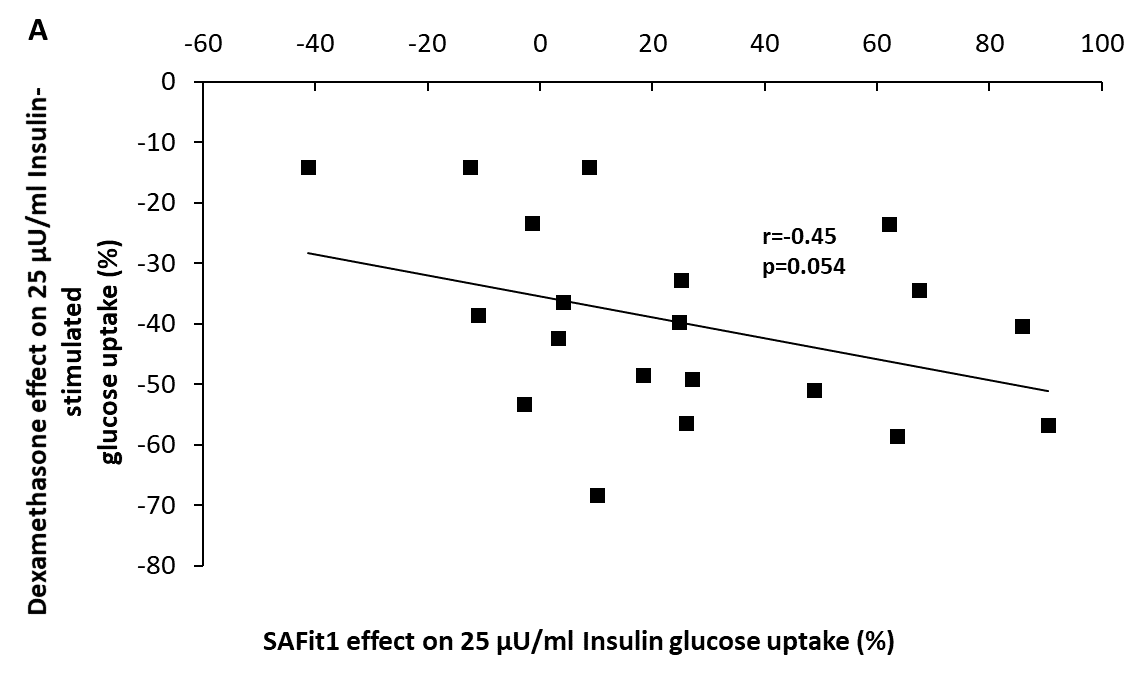
**

**
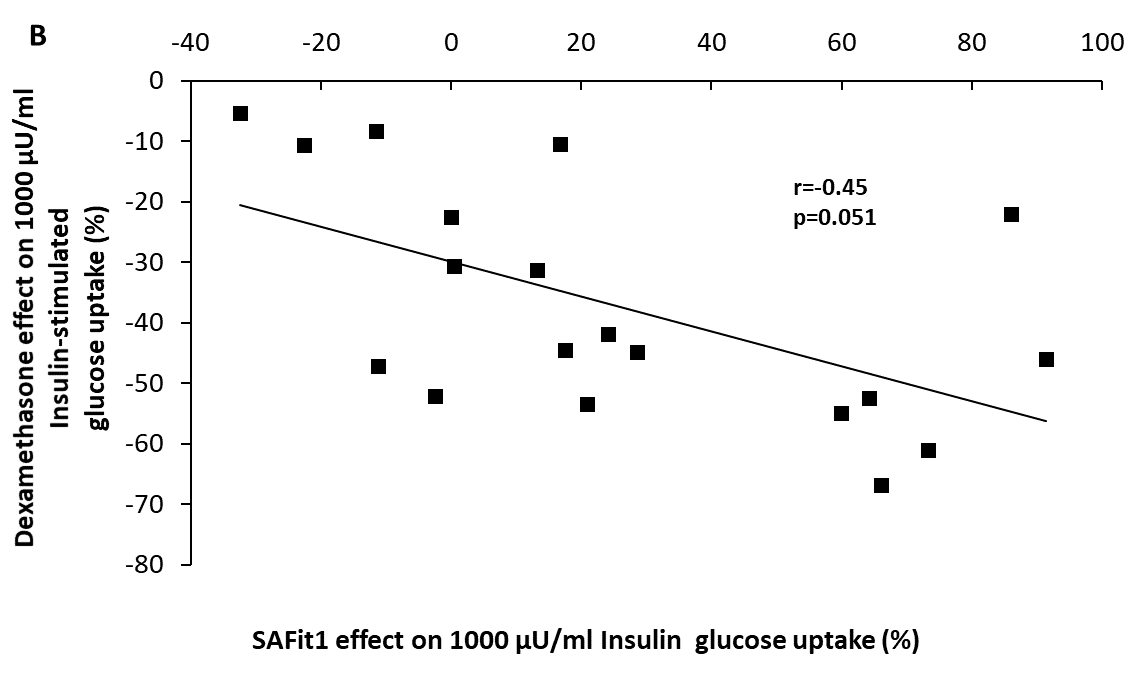
**

**Supplementary References**

1. Yu, Z.W., Jansson, P.A., Posner, B.I., Smith, U., Eriksson, J.W.: Peroxovanadate and insulin action in adipocytes from NIDDM patients. Evidence against a primary defect in tyrosine phosphorylation. Diabetologia **40**(10), 1197-1203 (1997). doi:10.1007/s001250050807

2. Dole, V.P., Meinertz, H.: Microdetermination of long-chain fatty acids in plasma and tissues. The Journal of biological chemistry **235**, 2595-2599 (1960).

3. Lundgren, M., Svensson, M., Lindmark, S., Renstrom, F., Ruge, T., Eriksson, J.W.: Fat cell enlargement is an independent marker of insulin resistance and 'hyperleptinaemia'. Diabetologia **50**(3), 625-633 (2007). doi:10.1007/s00125-006-0572-1

4. Pereira, M.J., Skrtic, S., Katsogiannos, P., Abrahamsson, N., Sidibeh, C.O., Dahgam, S., Månsson, M., Risérus, U., Kullberg, J., Eriksson, J.W.: Impaired adipose tissue lipid storage, but not altered lipolysis, contributes to elevated levels of NEFA in type 2 diabetes. Degree of hyperglycemia and adiposity are important. Metabolism (2016). doi:<http://dx.doi.org/10.1016/j.metabol.2016.09.008>

5. Pereira, M.J., Palming, J., Svensson, M.K., Rizell, M., Dalenback, J., Hammar, M., Fall, T., Sidibeh, C.O., Svensson, P.A., Eriksson, J.W.: FKBP5 expression in human adipose tissue increases following dexamethasone exposure and is associated with insulin resistance. Metabolism **63**(9), 1198-1208 (2014). doi:10.1016/j.metabol.2014.05.015

6. Lee, M.J., Fried, S.K.: Optimal protocol for the differentiation and metabolic analysis of human adipose stromal cells. Methods Enzymol **538**, 49-65 (2014). doi:10.1016/B978-0-12-800280-3.00004-9

7. Newell, F.S., Su, H., Tornqvist, H., Whitehead, J.P., Prins, J.B., Hutley, L.J.: Characterization of the transcriptional and functional effects of fibroblast growth factor-1 on human preadipocyte differentiation. FASEB J **20**(14), 2615-2617 (2006). doi:10.1096/fj.05-5710fje

8. Binder, E.B.: The role of FKBP5, a co-chaperone of the glucocorticoid receptor in the pathogenesis and therapy of affective and anxiety disorders. Psychoneuroendocrinology **34 Suppl 1**, S186-195 (2009). doi:10.1016/j.psyneuen.2009.05.021

9. Hauner, H., Schmid, P., Pfeiffer, E.F.: Glucocorticoids and insulin promote the differentiation of human adipocyte precursor cells into fat cells. The Journal of clinical endocrinology and metabolism **64**(4), 832-835 (1987). doi:10.1210/jcem-64-4-832

10. Hauner, H., Entenmann, G., Wabitsch, M., Gaillard, D., Ailhaud, G., Negrel, R., Pfeiffer, E.F.: Promoting effect of glucocorticoids on the differentiation of human adipocyte precursor cells cultured in a chemically defined medium. J Clin Invest **84**(5), 1663-1670 (1989). doi:10.1172/JCI114345

11. Ramirez-Zacarias, J.L., Castro-Munozledo, F., Kuri-Harcuch, W.: Quantitation of adipose conversion and triglycerides by staining intracytoplasmic lipids with Oil red O. Histochemistry **97**(6), 493-497 (1992).
