# Supplementary figures and images for: FKBP5 expression in human adipose tissue: potential role in glucose and lipid metabolism, adipogenesis and type 2 diabetes
Source: Endocrine. 2018 Jul 21;62(1):116–28. doi: 10.1007/s12020-018-1674-5 (PMC6153563; doi:10.1007/s12020-018-1674-5)

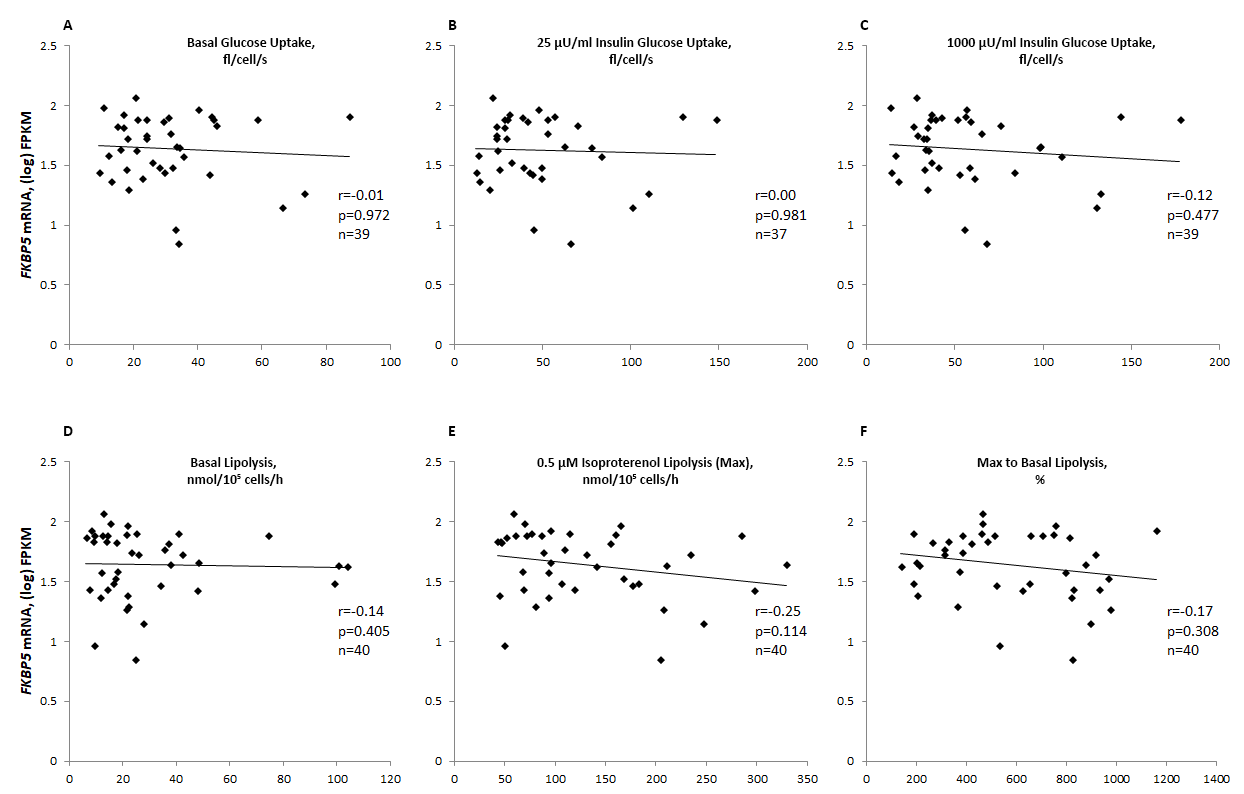

Supplement: Supplementary file 2 — Supplementary Figure1 [file 12020_2018_1674_MOESM2_ESM.tif]

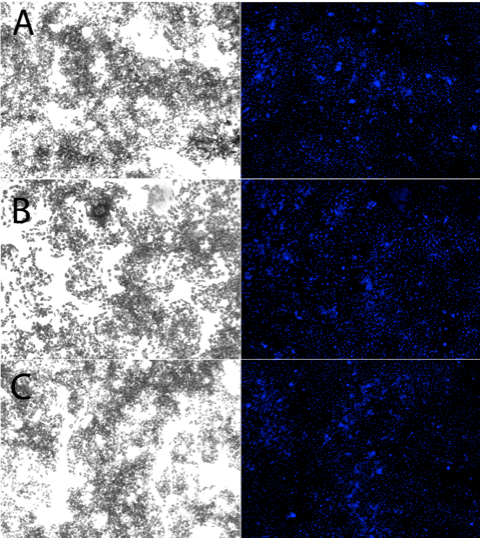

Supplement: Supplementary file 3 — Supplementary Figure2 [file 12020_2018_1674_MOESM3_ESM.tiff]

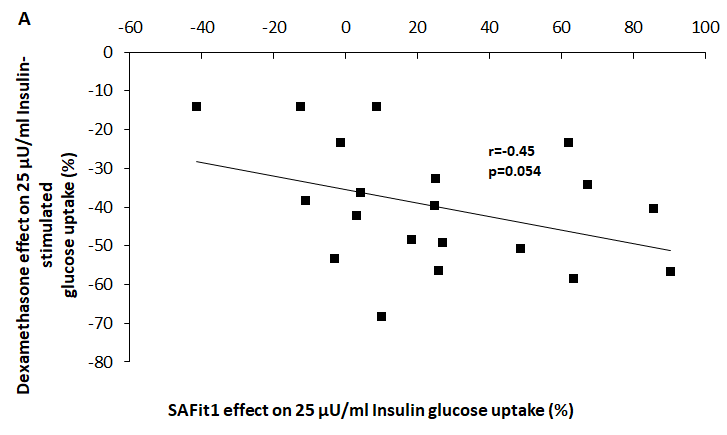

Supplement: Supplementary file 4 — Supplementary Figure3 [file 12020_2018_1674_MOESM4_ESM.tif]

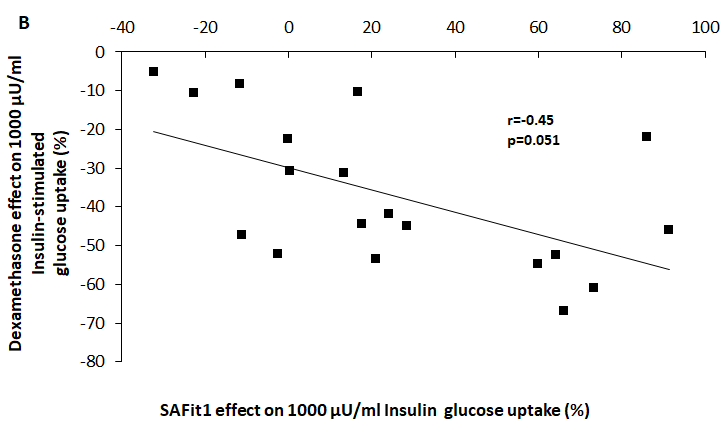

Supplement: Supplementary file 5 — Supplementary Figure4 [file 12020_2018_1674_MOESM5_ESM.tif]
